# Seasonal Antimicrobial Activity of the Airway: Post-Hoc Analysis of a Randomized Placebo-Controlled Double-Blind Trial

**DOI:** 10.3390/nu12092602

**Published:** 2020-08-27

**Authors:** Luis G. Vargas Buonfiglio, Oriana G. Vanegas Calderon, Marlene Cano, Jacob E. Simmering, Philip M. Polgreen, Joseph Zabner, Alicia K. Gerke, Alejandro P. Comellas

**Affiliations:** 1 Department of Internal Medicine, Carver College of Medicine Iowa City, University of Iowa, Iowa City, IA 52242, USA; luis-vargasbuonfiglio@uiowa.edu (L.G.V.B.); mcano@wustl.edu (M.C.); jacob-simmering@uiowa.edu (J.E.S.); philip-polgreen@uiowa.edu (P.M.P.); joseph-zabner@uiowa.edu (J.Z.); alicia-gerke@uiowa.edu (A.K.G.); 2 Department of Pediatrics, Carver College of Medicine Iowa City, University of Iowa, Iowa City, IA 52242, USA; oriana-vanegas@uiowa.edu

**Keywords:** humans, vitamin D, respiratory tract infections, peptides, antibacterial agents, double-blind method, seasons

## Abstract

Background: It is widely unknown why respiratory infections follow a seasonal pattern. Variations in ultraviolet B (UVB) light during seasons affects cutaneous synthesis of vitamin D_3_. Serum vitamin D concentration influences the expression of airway surface liquid (ASL) antimicrobial peptides such as LL-37. Objective: We sought to determine the effect of seasons on serum vitamin D levels and ASL antimicrobial activity. Methods: Forty participants, 18–60 years old, were randomized 1:1 to receive 90 days of 1000 IU vitamin D_3_ or placebo. We collected ASL via bronchoscopy and measured serum 25(OH) vitamin D from participants before and after intervention across seasons. We measured ASL antimicrobial activity by challenging samples with bioluminescent *Staphylococcus aureus* and measured relative light units (RLUs) after four minutes. We also investigated the role of LL-37 using a monoclonal neutralizing antibody. Results: We found that participants, prior to any intervention, during summer–fall (*n* = 20) compared to winter–spring (*n* = 20) had (1) decreased live bacteria after challenge (5542 ± 175.2 vs. 6585 ± 279 RLU, *p* = 0.003) and (2) higher serum vitamin D (88.25 ± 24.25 vs. 67.5 ± 45.25 nmol/L, *p* = 0.026). Supplementation with vitamin D_3_ increased vitamin D levels and restored ASL antimicrobial activity only during the winter–spring. The increased ASL antimicrobial activity seen during the summer–fall was abrogated by adding the LL-37 neutralizing antibody. Conclusion: ASL kills bacteria more effectively during the summer–fall compared to the winter–spring. Supplementation of vitamin D during winter–spring restores ASL antimicrobial activity by increasing the expression of antimicrobial peptides including LL-37.

## 1. Introduction

Respiratory infections are more prevalent during winter, contributing to excess hospital admissions and deaths [1,2,3,4]. However, we know little about why this seasonal pattern occurs. Several mechanisms have been proposed for the role of winter’s environment in increasing the likelihood of respiratory infections, such as crowding of infectious and susceptible hosts, increased replication of viruses, decreased mucociliary clearance, and impaired phagocytic activity of leukocytes [5,6,7]. 

Another possible reason why infections are seasonal is variations in serum vitamin D and its effect on the immune system [6]. Ultraviolet B (UVB) light converts 7-dehydrocholesterol in the skin into pre-vitamin D_3_ that spontaneously isomerizes into vitamin D_3_. In the liver, vitamin D_3_ from all sources is hydroxylated into 25(OH)D_3_, which is the storage form used to measure vitamin D_3_ sufficiency. Multiple sites, including the airway epithelia, can hydroxylate 25(OH)D_3_ into 1,25(OH)_2_D_3_ (calcitriol), which binds the vitamin D intracellular receptor and regulates cellular gene expression [8]. Exposure to sunlight during summer increases the concentration of vitamin D [9,10,11]. During winter, less exposure to UVB increases the likelihood of vitamin D_3_ deficiency, therefore potentially predisposing people to increased risk of developing respiratory infections [6,12]. In fact, one retrospective study found that vitamin D status had a negative and linear relationship with respiratory infections following a seasonal pattern [13].

The airway surface liquid (ASL) is rich in antimicrobial peptides and is one of the first lines of defense against pathogens. Impairment of ASL antimicrobial activity can result in respiratory infections [14]. Cathelicidin/LL-37 is one of the airway antimicrobial peptides. Its gene, Cathelicidin Antimicrobial Peptide (CAMP), has a vitamin D response element in the promoter region, which regulates its transcription [15,16]. Accordingly, we hypothesized that both vitamin D levels and ASL antimicrobial activity would follow a seasonal pattern. In addition, we hypothesized that vitamin D supplementation during winter–spring would restore decreased ASL antimicrobial activity to levels similar to those during summer–fall. We used the data from a previously published single-center, community-based, randomized, placebo-controlled, double-blind trial to investigate this hypothesis.

## 2. Materials and Methods

### 2.1. Study Design 

The methodology of this study has been previously published [17]. This single-center study investigated the effects of vitamin D_3_ supplementation on the innate immunity of the lung. The University of Iowa Hospital and Clinics Institutional Review Board approved the trial (IRB# 200607708). Written consent was obtained from all participants. This trial is registered on ClinicalTrials.gov (NCT01967628). 

### 2.2. Participants, Interventions and Randomization

Details of this study have been previously published [18]. Briefly, 105 participants, 18–60 years old, were recruited from Iowa City, IA, USA, at the University of Iowa. Exclusion criteria were as follows: unable to complete both visits, prior vitamin D supplementation within the previous three months, positive tuberculin skin test, pneumonia within the prior three years, any other airway infection or antibiotic in the last six weeks, or vaccination in the last month. Participants on prescription medications were excluded except for oral contraceptives, topical medications, selected antidepressants, levothyroxine, treatment for gastric reflux, antihistamines, and over-the-counter sleep aids. We did not include participants with asthma, diabetes, heart disease, or allergy to lidocaine (seven excluded). Pregnant women were excluded. After informed consent, 98 participants were randomized by a computer to receive 1000 IU of vitamin D_3_ or identical placebo capsules for 90 days on a 1:1 allocation ratio. ASL and blood were collected at baseline and post-intervention. Study participants, care provider, sponsoring agency, and researcher were blinded to assignments. Participants were excluded for this study if they were sick and unable to attend visits (*n* = 1), lost to follow-up (*n* = 1), or ASL was not collected due to failed bronchoscopy or less than 100 μg/mL of total protein (*n* = 56). Data were collected at The University of Iowa. The trial was stopped in July 2010. Analysis was done in original assigned groups. Baseline characteristics of the participants grouped by the seasons studied in the post hoc analysis are shown in Table 1.

### 2.3. Outcomes

The primary outcomes for this post hoc analysis were ASL antimicrobial activity and serum concentration of 25(OH) vitamin D_3_ (25(OH)D_3_) preintervention across seasons and postintervention (vitamin D_3_ or placebo). Secondary outcome for this analysis was the effect of treating ASL with an LL-37 neutralizing antibody in the preintervention ASL antimicrobial activity across seasons.

### 2.4. Adherence 

In the group of subjects that received vitamin D_3_, 39% of participants had residual doses, missing an average of 6.5 capsules (range 1–15), which represents on average only 7% of the total course of 90 days. In the group that received placebo, 53% missed an average of 8.8 capsules (range 1–30) [18].

### 2.5. Vitamin D Measurement

Blood samples were analyzed by the University of Iowa Department of Pathology Laboratory. 25(OH)D_3_ was measured by electrochemiluminescence multiplex flow immunoassay, and calcitriol was measured by quantitative chemiluminescent immunoassay.

### 2.6. Human ASL Samples

A pulmonary physician collected ASL via bronchoscopy under standard clinical protocol at the University of Iowa Hospitals and Clinics. A total of three sponges were placed, one at a time, on the right main stem bronchus or bronchus intermedius for 30 s each. Thereafter, sponges were eluted with 1 mL of isotonic saline, vortexed for 1 min, and stored at −80 °C. Protein concentration in the samples was measured using a Bradford assay. Each sample was adjusted to a concentration of 100 μg/mL of total protein, diluted with normal saline [18].

### 2.7. ASL Antimicrobial Activity

Bioluminescent *Staphylococcus aureus* Xen 29 was grown overnight using tryptic soy broth (TSB) media from a tryptic soy agar plate. Bacteria was diluted with a 10 mM pH 7.4 sodium phosphate buffer supplemented with 100 mM NaCl and 1% TSB to sustain bacterial luminescence, as previously described [19]. Ten microliters of protein-corrected ASL (100 μg/mL) was challenged with 10 μL of bioluminescent *Staphylococcus aureus* Xen 29 (~5 × 10^6^ colony forming units) into a 96-well plate at 37 °C. Relative light units (RLUs) were measured after 4 min. Conditions were randomized to the plate and uncovered after the results were available. 

### 2.8. Seasons and UV Intensity

Seasons are the result of variations in the intensity of solar radiation on the earth’s surface throughout the year. Since UVB in solar radiation is responsible for skin vitamin D_3_ photoconversion, we used daily measurements of UV Index from 2010 at solar noon in Iowa City, IA, USA. The radiation data were obtained from the National Aeronautics and Space Administration (NASA) Goddard Earth Sciences Data and Information Services Center (GES DISC). Surface UV exposure measurements are made by a spectrometer aboard NASA’s Earth Observing System’s Aura satellite, processed by the Finnish Meteorological Institute and archived at GES DISC [20].

We considered winter the months of December, January, and February; spring the months of March, April, and May; summer the months of June, July, and August; and fall the months of September, October, and November. Participants were assigned to the seasons based on the date of the bronchoscopy before intervention, when blood samples were also taken. 

### 2.9. LL-37 Antibody Assay

In a previous study, we determined that using a goat IgG LL-37 monoclonal antibody inhibited the antimicrobial activity of synthetic LL-37 in a concentration-dependent manner. This effect was not present when we treated samples with a control goat IgG targeting mouse IgG [18]. We preincubated ASL samples with the same LL-37 antibody (70 nM) for 1 h before antimicrobial activity assay. 

### 2.10. Statistical Analysis

Data are expressed as mean ± SEM. The Shapiro–Wilk test was used to determine the normality of the data. Unpaired Student’s *t*-test was used when comparing summer–fall vs. winter–spring and response to vitamin D vs. placebo by season. Fisher’s exact test was used for statistical comparison between categorical variables. ANOVA was used for statistical comparison between individual seasons. All data were analyzed using Graph Pad Prism^®^ 7.

## 3. Results

### 3.1. ASL Antimicrobial Activity Analyzed by Season

**Figure 1:**
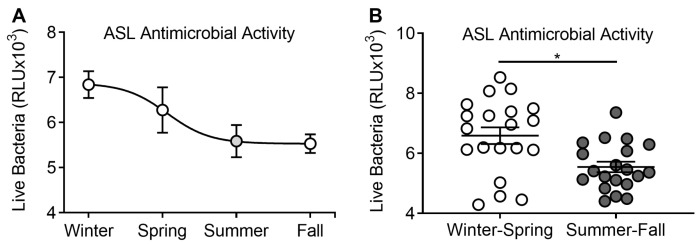
Seasonal distribution of airway surface liquid (ASL) antimicrobial activity. (**A**) Live bacteria after challenge with ASL collected during all seasons in relative light units (RLUs). Each circle represents the mean RLU from all challenges with ASL collected during that season. Decreased RLU was interpreted as increased antimicrobial activity. (**B**) Live bacteria after challenge with ASL collected during winter–spring compared to summer–fall. Each circle corresponds to the result of the challenge with ASL from one participant. * *p* < 0.05.

We investigated whether human ASL antimicrobial activity varied depending on the season when it was collected. We challenged bioluminescent bacteria with human ASL in vitro and measured RLU at four minutes to estimate remaining live bacteria [19]. We interpreted a reduction in RLU after challenge as increased antimicrobial activity. When we plotted the average of RLU per season in which bronchoscopy was performed, we observed that, indeed, remaining live bacteria after challenge with ASL followed a seasonal pattern (Figure 1A). We found significantly less live bacteria after challenge with ASL collected during summer–fall compared to the winter–spring seasons (5542 ± 175.2 vs. 6585 ± 279 RLU, *n* = 20 in each group, *p* = 0.003; Figure 1B). These results suggest that the airway’s intrinsic killing ability may vary with the season. 

### 3.2. Vitamin D_3_ Levels and Seasonal Pattern

**Figure 2:**
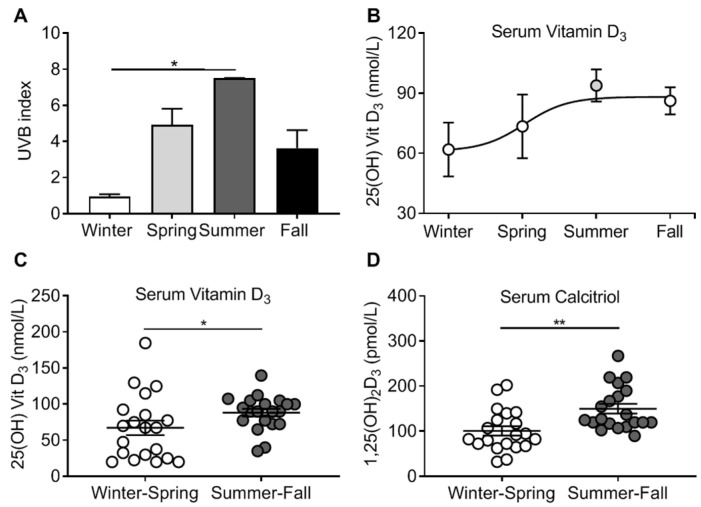
Seasonal distribution of serum vitamin D levels. (**A**) UVB index across all seasons. (**B**) Serum concentration of 25(OH) vitamin D of participants during all seasons. Each circle represents the mean serum concentration of 25(OH) vitamin D from all participants during that season. (**C**) Serum concentration of 25(OH) vitamin D of individual participants during winter–spring and summer–fall. Each circle corresponds to one participant. (**D**) Serum concentration of 1,25(OH) vitamin D (calcitriol) of participants during winter–spring and summer–fall. Each circle corresponds to the serum concentration of one participant. * *p* < 0.05, ** *p* < 0.01.

Vitamin D_3_ deficiency may increase the risk of respiratory infections, and in a prior study we demonstrated that ASL antimicrobial activity may be affected by serum vitamin D_3_ levels [18]. We considered that local variations in UVB during seasons influenced skin photoconversion to vitamin D_3_. Using NASA’s satellite database, we observed that the UVB index in the location of the study peaked in the summer and was lowest in the winter (Figure 2A). The serum concentration of 25(OH)D_3_ of participants also followed a seasonal pattern (Figure 2B). Serum 25(OH)D_3_ was significantly higher during summer–fall compared to winter–spring (88.25 ± 24.25 vs. 67.5 ± 45.25 nmol/L, *n* = 20 in each group, *p* = 0.026; Figure 2C). In addition, we found that serum concentration of calcitriol was also significantly higher during summer–fall compared to winter–spring (150.13 ± 10.75 vs. 101 ± 10.35 pmol/L, *n* = 20 in each group, *p* = 0.001; Figure 2D). These results suggest that both the storage and active forms of vitamin D_3_ vary with the seasons. 

### 3.3. Supplementation of Vitamin D_3_ during Winter–Spring, but Not during Summer–Fall, Improves ASL Antimicrobial Activity

**Figure 3:**
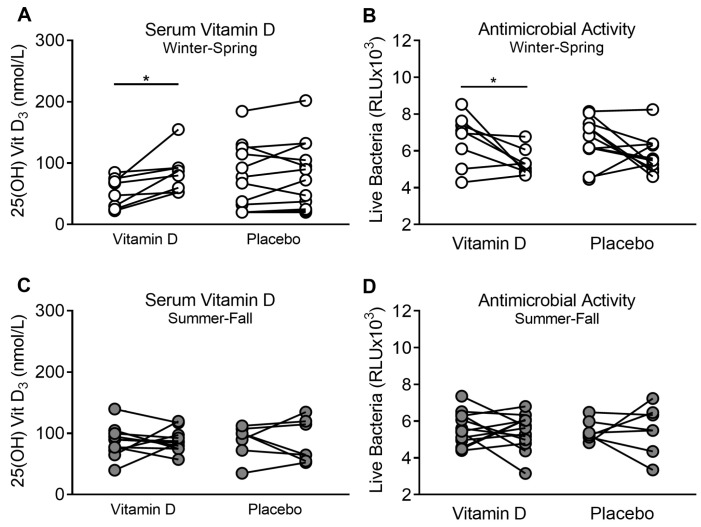
Serum vitamin D concentration and ASL antimicrobial activity in group supplemented with vitamin D. (**A**) Effect of supplementation of vitamin D vs. placebo on serum concentration of 25(OH) vitamin D during winter–spring. (**B**) Effect of supplementation of vitamin D vs. placebo on ASL antimicrobial activity during winter–spring. (**C**) Effect of supplementation of vitamin D vs. placebo on serum concentration of 25(OH) vitamin D during summer–fall. (**D**) Effect of supplementation of vitamin D vs. placebo on ASL antimicrobial activity during summer–fall. Each circle corresponds to one participant, and the lines connect the results before and after the respective intervention on the same participant. * *p* < 0.05.

We propose that the differences in antimicrobial activity are influenced in part by serum 25(OH)D_3_ concentrations. Since we found that during winter–spring serum 25(OH)D_3_ and ASL antimicrobial activity were lower, we hypothesized that supplementing vitamin D_3_ would increase the serum concentration of 25(OH)D_3_ and improve ASL antimicrobial activity. We found that participants who blindly received 1000 IU of vitamin D_3_ daily over 90 days during winter–spring increased their baseline serum 25(OH)D_3_ concentrations (52.83 ± 8.75 vs. 84.08 ± 11.78 nmol/L, *n* = 8, *p* = 0.015; Figure 3A), and ASL decreased the number of live bacteria after challenge (6625 ± 496.9 vs. 5362 ± 252.8 RLU, *n* = 8, *p* = 0.04; Figure 3B).

When participants were supplemented with placebo, we found no significant difference from their baseline in live bacteria after challenge and serum levels of vitamin D (Figure 3A–D). Similarly, we found that both vitamin D serum levels and antimicrobial activity of participants who received vitamin D_3_ during winter–spring were not significantly different when compared to participants during summer–fall before any intervention (5362 ± 252.8 vs. 5542 ± 175.2 RLU, *p* = 0.56; 35.3 ± 2.16 vs. 33.63 ± 4.71 ng/mL, *p* = 0.71, *n* = 8 and 20, respectively), suggesting that oral supplementation restores vitamin D serum levels and antimicrobial activity similarly to sunlight exposure. These results suggest that the seasonal antimicrobial activity of the airways might be related to serum vitamin D concentration.

We also found that supplementing with vitamin D_3_ during summer–fall had no significant effect in both serum vitamin D levels and ASL antimicrobial activity (Figure 3C,D).

### 3.4. LL-37 Antibody Abrogates Seasonal Differences in ASL Antimicrobial Activity

**Figure 4:**
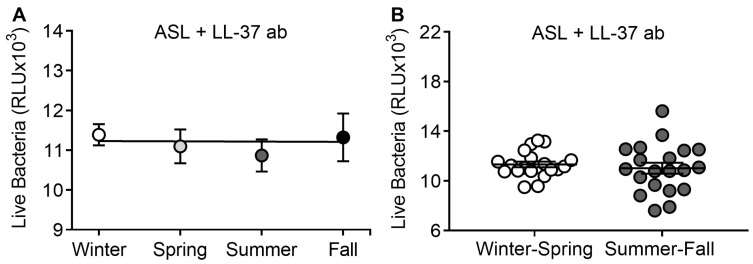
ASL treated with an LL-37 antibody abrogates seasonal antimicrobial activity. (**A**) Antimicrobial activity of ASL neutralized with an LL-37 antibody during all seasons. Each circle represents the mean RLU from all challenges with neutralized ASL collected during that season. (**B**) Antimicrobial activity of ASL neutralized with an LL-37 antibody grouped by winter–spring and summer–fall. Each circle corresponds to one participant.

Since vitamin D increases the transcription of cathelicidin/LL-37 in the airways [16], we hypothesized that enhancement of antimicrobial activity during summer–fall was related to LL-37 activity. We have previously shown that using an LL-37 neutralizing antibody dampens the antimicrobial activity of ASL [18]. We found that by adding LL-37 antibody to ASL, the difference between winter–spring and summer–fall was no longer present (11326 ± 230.9 vs. 11016 ± 440.1 RLU, *p* = 0.54, *n* = 20 in each group; Figure 4A,B). This result suggests that LL-37 might be implicated in seasonal antimicrobial activity of the airways. 

## 4. Discussion

To our knowledge, this is the first study showing that human ASL antimicrobial activity may have a seasonal pattern that matches with the rise and fall in respiratory infections in the northern hemisphere. 

Seasons are the product of variations in solar radiation throughout the year. It has been hypothesized that, similarly to many other seasonal biological processes, respiratory infections might be influenced by solar radiation [1]. A potential link between solar radiation and respiratory infections is that UVB in sunlight increases skin photoconversion of vitamin D and affects airway immunity by increasing ASL antimicrobial peptides such as LL-37. We found that summer–fall compared to winter–spring had (1) a higher combined total UV index, (2) higher levels of serum vitamin D, and (3) higher ASL antimicrobial activity. 

A previous retrospective study also showed that vitamin D status was seasonal and had a negative, linear relationship with the risk of respiratory infections. The authors suggested that prospective randomized trials were warranted to investigate the role of vitamin D supplementation to establish the underlying mechanisms [13]. We analyzed the effect of vitamin D supplementation during different seasons and found that participants supplemented with vitamin D during winter–spring increased both their baseline vitamin D levels and ASL antimicrobial activity to similar values present in participants during summer–fall without supplementation. Furthermore, summer–fall supplementation of vitamin D did not increase vitamin D levels or ASL antimicrobial activity. We reason that this is related to participants’ vitamin D status before supplementation. 

We observed that serum vitamin D did not exactly correlate with levels of UVB indexes. Vitamin D levels are not only influenced by the current UVB index of the season, but also by UVB exposure from the prior season to when the sample was taken. Therefore, the peak of UVB seen during summer will likely last over the fall and have its nadir during winter. This effect was reported in a cohort where subjects were vitamin D sufficient by the end of summer, and more than half of the participants were insufficient by the end of winter. Accordingly, there is a potential role for vitamin D supplementation during these months of low UVB [9].

A recent meta-analysis of human clinical trials found that vitamin D supplementation prevented respiratory infections in individuals with severe deficiency, but no further benefit was seen with normal values [21]. Most of our participants receiving vitamin D during winter–spring had vitamin D levels that were below 75 nmol/L and increased their levels on average above this level after supplementation. Although the subjects were not deficient (<50 nmol/L), we consider that the subjects can be considered insufficient (50–75 nmol/L) [9]. We propose that a suboptimal level of serum vitamin D during winter–spring may be a contributing factor for impaired antimicrobial activity of the airways. In addition, no benefit was seen by supplementing during the summer when levels were optimal on average. This study provides a rationale for testing whether supplementing with vitamin D during winter–spring is enough to alter the seasonal pattern of respiratory infections.

It has been hypothesized that LL-37 via vitamin D induced expression may contribute to the seasonality of airway infection; however, this has never been proven. In this study we used an indirect approach to investigate the role of LL-37 in seasonal ASL antimicrobial activity. We treated samples with an LL-37 antibody. We observed that the difference between seasons was abrogated, suggesting that LL-37 likely plays a role in the seasonality of ASL antimicrobial activity. 

Our study has several limitations, including (i) data are from a small RCT in which groups were not originally stratified by season; (ii) the vitamin D and ASL antimicrobial activity are based on a single measure; (iii) levels of physical activity, time indoors, or dietary intake of vitamin D were not collected; (iv) participants were mainly young, thus we were not able to determine the significance of our findings in an older population; (v) limited volume and concentration of the ASL samples collected restricted our ability to directly measure LL-37 concentrations; (vi) although the difference in live bacteria reflects difference in pathogen inoculum, which is an important factor in the development of infection, our study did not assess antibacterial activity and inactivation LL-37 in vivo, thus the clinical significance is uncertain; (vii) we only tested one bacterial species, *S. aureus*, which served as a model to test ASL antimicrobial activity. Although we do not know the generalizability of our findings to other pathogens, we consider that our results can be viewed as a proof of principle, and that, more than likely, it is possible these results explain a portion of seasonable variability in respiratory infections. 

## 5. Conclusions

We propose that lower levels of vitamin D during low UV index seasons impairs the antimicrobial activity of the airways, likely by decreased ASL antimicrobial peptides such as LL-37, increasing the susceptibility to respiratory pathogens. Larger studies designed to address all of the above limitations are needed to confirm whether supplementation alone of Vitamin D during these seasons is sufficient to prevent seasonal respiratory infections.

## Figures and Tables

**Table 1:** Comparison of patient characteristics at baseline by treatment group.

Subject Characteristics	Summer–Fall	Winter–Spring	*p* Value
Number of participants	20	20	…
Age (years)	28.9 (19–51)	26.15 (19–60)	0.41
Male/female sex (%)	55/45	75/25	0.32
Ethnicity (% white)	100	80	0.11
Smokers (%)	30	30	0.21
Pack/years	24.67 (10–25)	16.75 (10–45)	0.24

Data expressed as mean and 95% CI.
